# Meeting of Dec. 21, 1874

**Published:** 1875-01-01

**Authors:** 


### REGULAR MEETING, DECEMBER 21, 1874.

DR. F. C. HOTZ read the following report of experiments made by Drs. Prœgler, Wild, and himself, during the past summer on the

### TRANSFUSION OF LAMB'S BLOOD IN THE HUMAN SUBJECT.

Over two centuries ago the transfusion with blood of an animal into human beings had been practiced with the idea of invigorating the broken-down constitution of invalids. But the hopeful expectations that at last the secret of prolonging life had been discovered, were sadly disappointed. The operation failed, and for two hundred years it rested in peace, to be revived once more, and to gain some notoriety again by Dr. Oscar Hasse, (Nordhausen, Germany) who in the early part of this year published an account of fifteen cases of direct transfusion of lamb's blood ("Die Lammblut Transfusion beim Menschen", von Dr. O. Hasse, Leipzig, 1874). He transfused seven times for phthisis, with five good results and two failures; four times successfully for chronic anæmia, and once for severe hæmorrhage with placenta præria; once for cachexia from caries with temporary improvement of the patient; once for carcinoma without benefit, and once in a case of paralysis agitans, which died five hours after the operation, under the symptoms of cerebral apoplexy.

Summing up his results, Dr. Hasse says, on page 62: "As to the greatest gain, I would like to call the attention to the most material aid which we have found the direct transfusion of lamb's blood to give us against phthisis. For just in these cases the success was simply wonderful; the general health of these patients was soon perfectly satisfactory, and the local disease continued to improve steadily, as confirmed by the physical examination."

So enthusiastic a statement apparently based upon facts, could not fail to make an impression on the medical fraternity, and to induce others to try the operation. The fiasco it had met with in former times, could not be a reasonable objection to a new trial, for do we not flatter ourselves with having improved since that time, in our knowledge of human anatomy and pathology, as well as in surgical operations and instruments? Are not other operations (e. g., for catarrh) which in former days achieved the most pitiful results, now the brightest stars of surgical art? Perhaps the same was the case with the transfusion, and if it proved as efficient in arresting pulmonary phthisis as Dr. H. claimed it to be, it would certainly be the most valuable acquisition. A priori, it is true, we could not understand how the transfused blood was to act upon the pulmonary disease. It did not seem very likely that the simple addition to the blood of a few ounces of fresh lamb's blood, would materially influence so complicated a trophic disorder as the phthisis is the result of. But we are daily using a great many remedies, because by experience we know them to be useful, although we cannot comprehend their action fully yet. At the present time we do not decide upon the merits of new therapeutics by theoretical speculations, but by experiments. This method only could determine as to the value of Dr. Hasse's operation, and the great importance attached to any question referring to the treatment of phthisis, demanded a careful and thorough investigation. Such was made during the summer by Drs. Prœgler, Wild, and myself, performing nine transfusions with lamb's blood. I shall presently give you the results of our operations; but first, let me say a few words about the modus operandi.

Our method of operating did not differ from Hasse's materially; a few modifications were introduced to simplify the operation. A young healthy lamb was securely strapped on a suitable board, the wool removed from one side of the neck, and the carotid artery exposed to at least one and one-half inches in length. At the central end (*i. e.*, towards the heart,) a small clamp was applied for temporarily interrupting the circulation, while at the peripheric side (*i. e.*, towards the head,) the artery was permanently closed by a ligature. The piece of the carotid thus included between the ligature and clamp was then opened sufficiently to allow the introduction of the bulbous point of a glass tube, to the other end of which a piece of india-rubber tube, about three inches in length was attached. After the opened artery was carefully cleansed of any coagula, the glass tube was tied in. Leaving the lamb then for a few minutes, we turned our attention to the patient. Sitting in a chair, he put his arm on a table; a subcutaneous vein of suitable size was selected, exposed by a sufficient incision, and ligated at the peripheric angle, while compressed with the finger at the central angle; the intermediate piece opened, a silver canula was inserted far enough, as to be held firmly by the elastic wall of the vessel. For this purpose the silver tube was made very slender and slightly conical. At this moment the lamb was placed on the table with its neck opposite the patient's arm; the compression of the vein discontinued, a few drops of blood would fill the silver canula by reflux, and at this very instant the clamp was removed from the carotid artery of the lamb; and when the arterial blood almost instantaneously filling the glass tube and rubber was discharged from the mouth of the rubber tube, this had to be nicely connected with the silver canula. In order to facilitate this very important manipulation, we attached to the rubber a short conic nozzle, which nicely fitted in the funnel-shaped mouth of the silver tube. By this means the connection could be established quickly and surely while the blood was flowing, and all danger of air entering the vein was obviated.

The regular symptoms recorded during the transfusion, were a sensation of warmth in the arm, a rapidly increasing redness of the face, perception of snowflakes, dizziness, nausea and vomiting, pain in the back, and dyspnœa, which finally increased to such a degree as to necessitate the interruption of the transfusion.

From the first moment that the blood began to enter the vein, our assistant took an accurate account of the time; in our cases the transfusion lasted from eighty to one hundred and twenty seconds. The operation over, the vein was closed by digital compression, the silver tube in connection with the rubber withdrawn, and the lamb's blood allowed to flow into a glass for ten seconds more, in order to ascertain by this quantity the total amount of transfused blood.

Then the central end of the carotid was ligated, the whole apparatus removed, and the wound of the lamb's neck united by a few stitches. The patient's wound was dressed simply with wet lint, and in all cases healed favorably.

About one-half to one hour after the operation, the patients were thrown into a violent rigor, lasting fully an hour, after which they felt greatly exhausted, and fell into a sound sleep.

The first urine passed after the transfusion, was more or less bloody in six cases, and in three cases, the sputa were also tinged with blood. But the urticaria which Dr. Hasse observed almost regularly, appeared in one case only.

And now, gentlemen, I will give you a brief report of our cases, stating the status quo anté, and a few months after the transfusion.

CASE 1.—Mr. Kl——, aged thirty-two, a German teacher, had been suffering from pulmonary disease for over two years; anæmic, pretty emaciated, coughing frequently, and raising a copious amount of puriform sputa; no appetite. Over the apex of the lungs, in the first and second intercostal space, dullness of percussion, and bronchial breathing with moist rales; the lower portion of the lungs normal. On the thirtieth of May, transfusion of eighteen ounces of lamb's blood; six days later, urticaria over his whole body; during June and July, better appetite, improved general appearance and physical strength. The cough however did not abate, and the patient gradually grew worse again. An examination made October 15th, confirmed a decided progress of the disease, the consolidation of the lungs extending to the fourth rib on the right, and to the fifth rib on the left side.

CASE 2.—H. K., aged thirty-seven, butcher, suffering from "asthma" and cough for five years; repeated hæmorrhage from the lungs within the last three years, no appetite, slight emaciation; right lung normal, upper part of left lung consolidated, in lower portion numerous rales; muco-purulent sputa, feeble pulse. The transfusion of eighteen ounces of lamb's blood (June 9th,) was followed by an improved appetite, and better feeling; he spent the summer and fall on a farm, and returned home this month greatly emaciated, with a dry frequent cough, and the tubercular deposits in the left lung extending down as far as the angle of the scapula.

CASE 3.—Mrs. W., aged thirty-two, in seventh month of pregnancy, was so anæmic, as to faint several times during one day; pulse exceedingly thin, urine contained slight traces of albumen. The transfusion of sixteen ounces of lamb's blood (June 16th,) did not even temporarily benefit the patient; her urine showed large amount of albumen for several weeks following the operation.

CASE 4.—N. W., aged thirty-seven, laborer, in the last stage of consumption; extensive cavities in the right lung, and consolidation of the upper half of the left lung; dyspnœa, extreme emaciation, night sweats, copious discharge of greenish yellow sputa, no appetite, no sleep. June 21st, transfusion of sixteen ounces of lamb's blood, followed by an improvement of appetite, and subjective feeling; on July 31st, the state of the lungs was unchanged, and since that time the patient was lost sight of.

CASE 5.—C. F. V., aged forty, cigar dealer, opium eater and consumptive; exceedingly emaciated; feeble and quick pulse; sallow complexion; copious expectoration of greenish yellow sputa; cavity in the upper half of the left lung, and consolidation of the lower half, with an ill-defined respiratory murmur; also, tubercular infiltration of the upper part of the right lung as far as the fourth rib. Sixteen ounces of lamb's blood were transfused on July 1st, and a week later a striking improvement of his complexion and appetite could be noticed; but did not last long. An ultimate examination made October 17th, discovered a marked progress of the tubercular disease; had lost five pounds since operation.

CASE 6.—Fr. H., aged twenty-nine, blacksmith, was taken with pleuro-pneumonia in spring, and laid up ever since. No appetite; weak, feverish, emaciated, constantly coughing and raising a large amount of puriform sputa. The respiratory movements of the left side were very faint, and all over the left lung there was a dull percussion sound, with loud bronchial respiration in the upper half, and remote ill-defined murmurs below the fourth rib. Right lung good. After fifteen ounces of lamb's blood had been transfused, (August 1st,) the patient slowly improved, so that by October 22nd, he had gained eight pounds in weight, coughed very seldom; the sputa were scanty and simply mucous; breathing easier, the expansion of the left lung stronger; the dullness and bronchial respiration limited to the posterior upper part of the left lung.

CASE 7.—Chas. H., aged thirty-eight, farmer, very anæmic, complaining of palpitation of the heart, sleeplessness, cold feet, and inability to do any work; no organic disease could be found. On August 22nd, he received twenty ounces of lamb's blood by direct transfusion; the urine first bloody, afterward albuminous had to be drawn by means of the catheter for seven days. Patient complained of headache, dizziness, numbness of hands, and was unable to walk; he gradually failed, and died three weeks after the operation. By a post mortem examination, an acute inflammation of the kidneys, and a great number of small hæmorrhagic spots all over the brain, and in the dura mater were found.

CASE 8.—Mrs. K., aged ——, had a very severe hæmorrhage four weeks after a forceps delivery. After the bleeding was successfully arrested, twelve ounces of lamb's blood were transfused (August 30th,) into the patient, whose limbs were cold and pulseless. She at once revived, and by a liberal diet, made a quick recovery.

CASE 9.—N. W., aged thirty-two, farmer, anæmic and weak, so much so, that walking across a room exhausted him completely; except a slight dullness over the apex of both lungs, nothing abnormal could be discovered. A transfusion of eighteen ounces of lamb's blood (August 15th,) produced a marked improvement; he grew stronger, so as to take long walks. But two months later he began to fail again, and gradually relapsed into the status quo ante.

The above statement shows that we have operated five times for phthisis, three times for chronic anæmia, and once for hæmorrhage, with this result: that the operation was a failure in six cases, (1, 2, 3, 4, 5, 9); it caused the death of one patient, (7), and two patients only (6 and 8) were permanently benefited. Our experiments, therefore, do not corroborate Dr. Hasse's observations, although some of our patients offered the most favorable conditions for a good result (cases of simple anæmia). All the patients experienced an improvement of their feeling and appetite, for the four to six weeks following the transfusion. This temporary benefit, together with the good result obtained in the case of hæmorrhage, might speak in favor of performing the operation in cases of profuse loss of blood. Here an instantaneous supply of blood will remove the immediate danger, and allow the body to reproduce its own blood by the time the transfused lamb's blood is exhausted. But will a lamb, or any other suitable animal be at our disposal, just when we want it in such cases of emergency? shall we not find it easier in such cases to get a few ounces of human blood, and to perform the indirect transfusion?

But there are other facts, which just as strongly as our experiments, speak against the probability of permanently benefiting human beings by a transfusion of animal blood.

PROF. LANDOIS, (Greifswald, Germany,) transfusing one animal with the blood of a different species, (e. g., rabbits with the blood of sheep), found that the serum of the blood of one animal dissolved the blood corpuscles of other animals; portions of the blood taken at different intervals after transfusion, showed the corpuscles in all stages of dissolution; the hæmoglobin of the destroyed cells was disposed of principally by the kidneys (*Am. Jour. Med. Sci.*, Oct., 1874). And this accounts for the so-called "bloody" urine discharged after transfusion.

PROF. PONFICK, (Rostock, Germany,) could examine the blood of a woman who died twenty minutes after the transfusion of lamb's blood. He found her blood full of small yellowish elements, the largest of which were two-thirds the size of human blood-cells, and then there were smaller ones of all grades down to the finest granules. The shape, color, and chemical reaction characterized these bodies as blood-cells on a reduced scale; besides these, there was the regular number of normal human blood corpuscles, which were larger by one-third than the lamb's blood cells. Debris of red corpuscles were found enclosed in the white cells (*Berl. Klinische Wochenschrift*, 1874, No. 28).

What Landois demonstrated on animals, Ponfick's observation thus fully verified on the human blood; the introduction of foreign blood leads to a dissolution of red corpuscles, and most likely of those of the foreign blood, and the material of the destroyed cells simply serves as food for the white blood corpuscles. This accumulation of nutritive material in the blood, is the only way by which the transfusion of animal blood can benefit a human subject, and it also accounts for the temporary relief our patients have experienced shortly after the operation. But as there is no probability that the foreign blood cells will ever assume the physiological properties, and discharge the physiological functions of human blood corpuscles, it is impossible to permanently replace any want of human blood by the transfusion of lamb's blood.

DISCUSSION.

The subject of the paper was then briefly commented upon by Drs. Paoli, Adolphus, Earle, and others.

At the request of the society, Dr. Gradle (Interne Physician at Mercy Hospital), made a short statement regarding a case of supposed catalepsy, now in the Mercy Hospital. Several members present who had seen the case, expressed their views regarding it. Dr. Hotz thought the patient was insane, and only feigning unconsciousness. Dr. Earle considered it a case of Hysteria.

On motion, the society adjourned.

